# Serum zinc levels in 368 patients with oral mucosal diseases: A preliminary study

**DOI:** 10.4317/medoral.21079

**Published:** 2016-03-31

**Authors:** Zhe-Xuan Bao, Xiao-Wen Yang, Jing Shi, Li-Xin Liu

**Affiliations:** 1 First clinical medical school of Shanxi Medical University, Taiyuan, Shanxi, China; 2 Department of Oral Medicine, Shanxi Provincial People’s Hospital, Taiyuan, Shanxi, China; 3 Department of Hospital Infection Control, Shanxi Provincial People’s Hospital, Taiyuan, Shanxi, China; 4 Department of Gastroenterology and Hepatology, The First Clinical Hospital of Shanxi Medical University, Taiyuan, Shanxi, China

## Abstract

**Background:**

The aim of this study was to assess the serum zinc levels in patients with common oral mucosal diseases by comparing these to healthy controls.

**Material and Methods:**

A total of 368 patients, which consisted of 156 recurrent aphthous stomatitis (RAS) patients, 57 oral lichen planus (OLP) patients, 55 burning mouth syndrome (BMS) patients, 54 atrophic glossitis (AG) patients, 46 xerostomia patients, and 115 sex-and age-matched healthy control subjects were enrolled in this study. Serum zinc levels were measured in all participants. Statistical analysis was performed using a one-way ANOVA, t-test, and Chi-square test.

**Results:**

The mean serum zinc level in the healthy control group was significantly higher than the levels of all other groups (*p* < 0.001). No individual in the healthy control group had a serum zinc level less than the minimum normal value. However, up to 24.7% (13/54) of patients with AG presented with zinc deficiency, while 21.2% (33/156) of patients with RAS, 16.4% (9/55) of patients with BMS, 15.2% (7/46) of patients with xerostomia, and 14.0% (8/57) of patients with OLP were zinc deficient. Altogether, the zinc deficiency rate was 19.02% (70/368) in the oral mucosal diseases (OMD) group (all patients with OMD). The difference between the OMD and healthy control group was significant (*p* <0.001). Gender differences in serum zinc levels were also present, although not statistically significant.

**Conclusions:**

Zinc deficiency may be involved in the pathogenesis of common oral mucosal diseases. Zinc supplementation may be a useful treatment for oral mucosal diseases, but this requires further investigation; the optimal serum level of zinc, for the prevention and treatment of oral mucosal diseases, remains to be determined.

**Key words:**Oral mucosal diseases, Zinc deficiency, pathogenesis.

## Introduction

Oral mucosal diseases (OMD) are common disorders affecting all segments of the general population that represent a series of abnormal alterations in oral mucosal surfaces, which include the labial and buccal mucosae, the alveolar mucosa, all parts of tongue, the floor of the mouth, and the gingival, palatal, and oropharyngeal mucosae (1,2). Although the majority of OMDs are benign, some of them are difficult to manage and usually interfere with important oral functions, systemic health, and quality of life of the patients.

Recurrent aphthous stomatitis (RAS), oral lichen planus (OLP), burning mouth syndrome (BMS), atrophic glossitis (AG), and xerostomia are some of the most prevalent oral mucosal diseases and they typically require multiple visits to specialist outpatient services. However, the etiology and pathogenesis of these disorders is still poorly understood and highly debated (2-5).

**Figure 1:**
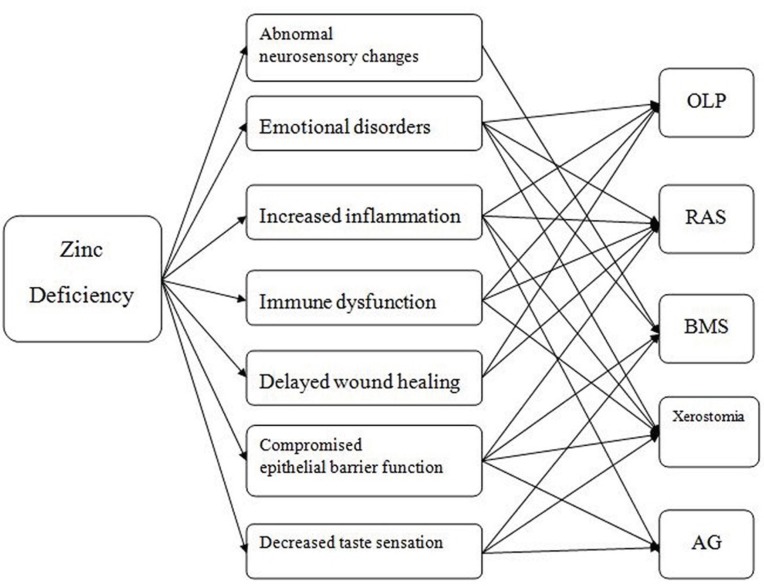
Zinc deficiency may be involved in the pathogenesis of common oral mucosal diseases through a variety of mechanisms. OLP: oral lichen planus. RAS: Recurrent aphthous stomatitis. BMS: burning mouth síndrome. AG: atrophic glositis.

Zinc, one of the most important trace elements within the human body, plays a variety of critical roles in cell growth and reproduction, normal immune functions, collagen synthesis, and wound healing (6,7). And zinc deficiency is related to multiple physiological abnormalities. Therefore, it is plausible that zinc deficiency might be directly involved in the pathogenesis of some oral mucous diseases (Fig. 1). At the present, the available literature concerning serum zinc levels in patients with oral mucosal diseases is severely limited. Moreover, findings from several of these studies are inconsistent (4,8-14) ( Table 1), which demonstrates that further research is needed.

**Table 1:**
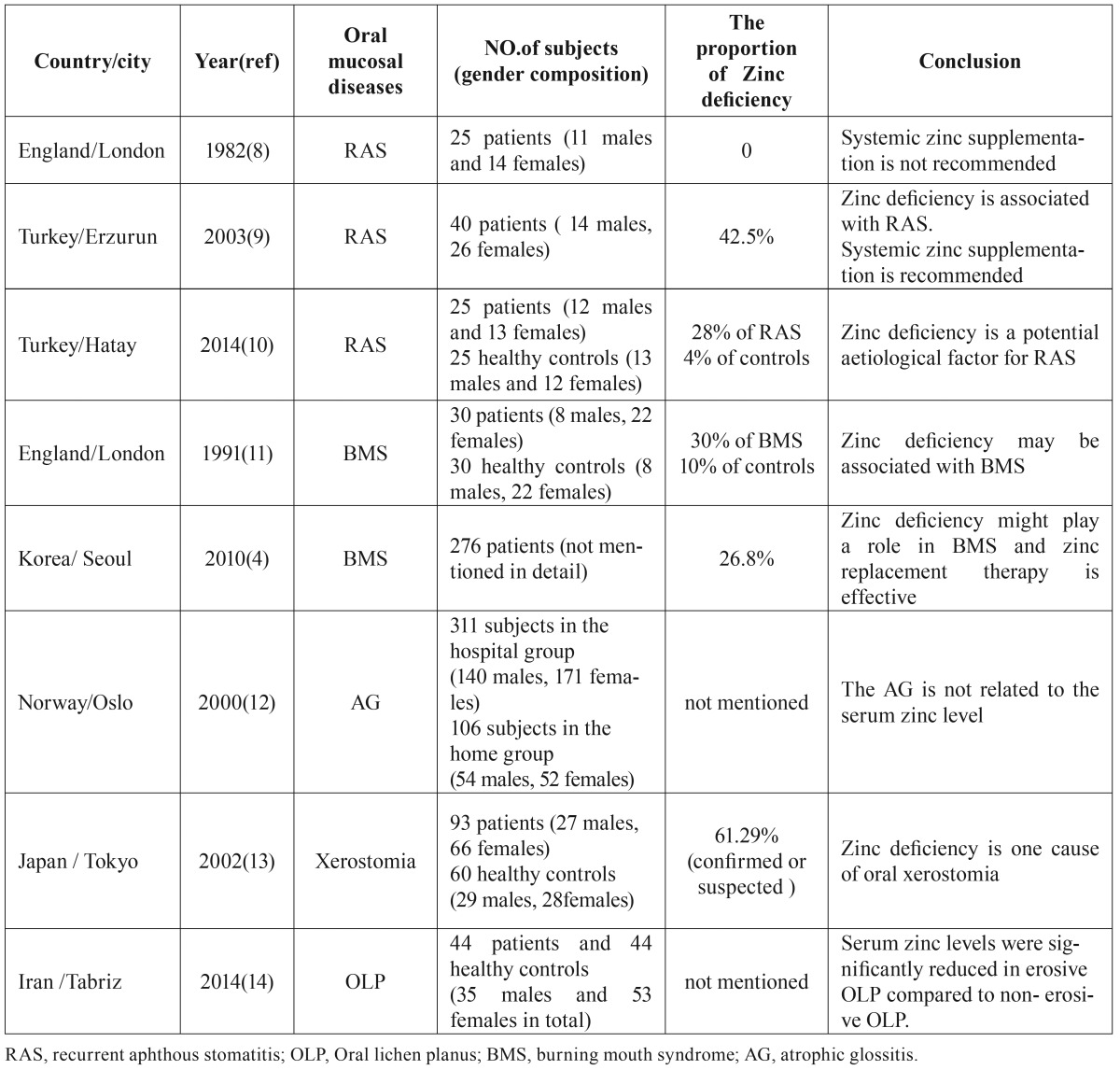
Existing reports of zinc deficiency in oral mucosal diseases in the past three decades.

The aim of this study was to assess the serum zinc levels of patients with some of the most common oral mucosal diseases as compared to healthy controls to determine if zinc deficiency may play a role in the etiology and pathogenesis of oral mucosal disease.

## Material and Methods

A total of 368 patients, consisting of 156 RAS patients, 57 OLP patients, 55 BMS patients, 54 AG patients and 46 xerostomia patients were enrolled in this study. Sex- and age-matched healthy control subjects were also examined (115 total controls). All participants were evaluated from January 2013 to May 2015 at the Department of Oral Medicine, Shanxi Provincial People’s Hospital, China. An OMD diagnosis was established according to accep-ted criterions (1,4,13-20) ( Table 2). Other less common diseases, such as oral leukoplakia (OLK), discoid lupus erythematosus (DLE), oral acute infection, allergic reactions, and seasonal disorders, such as cheilitis, were not included in this study. None of the control subjects had any oral mucosal lesions or related systemic diseases.

**Table 2:**
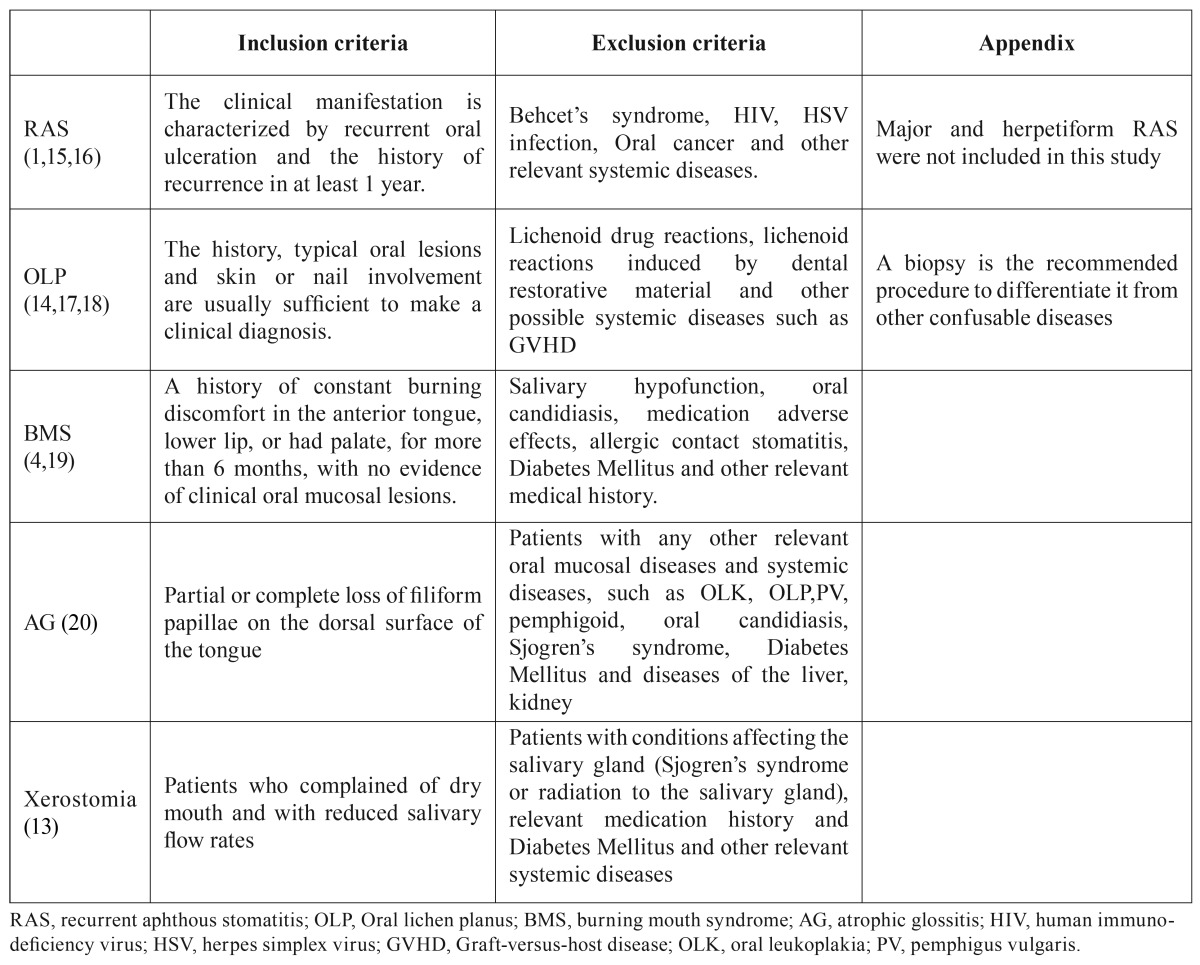
Diagnostic criteria for the oral mucosal diseases involved in the present study.

For all participants, blood samples were taken after 12 hours of overnight fasting, medical histories were recorded in detail, and a thorough clinical oral examination was performed by the same professional dentist. The study was approved by the hospital ethics committee and informed consent was obtained from each participant.

Serum zinc levels were measured using a Colorimetric Method Zinc Assay Kit (BSBE, Beijing, China). The accepted normal serum zinc level is 10.7-17.70 μmol/L. Zinc deﬁciency was deﬁned as a serum Zn level below this cutoff.

Statistical analysis was performed using SPSS version 18.0 software (Chicago, Illinois, USA). Serum zinc levels were compared using a t-test to compare between each OMD group and the healthy control group, while one-way ANOVA was used to compare among different groups. In each group, serum zinc levels were compared between male and female using a t-test. In addition, the sex ratio was compared for all groups by Chi-square test. A *p*-value < 0.05 was accepted as statistically significant.

## Results

- The serum zinc levels 

Although the serum zinc levels of some OMD patient groups were still in normative range, the mean serum zinc level of all patients with any OMD was significantly lower than that of the control group (*p* <0.001,  Table 3). Each individual in OMD group had a lower mean serum zinc level than the control group (*p* <0.001).

**Table 3:**
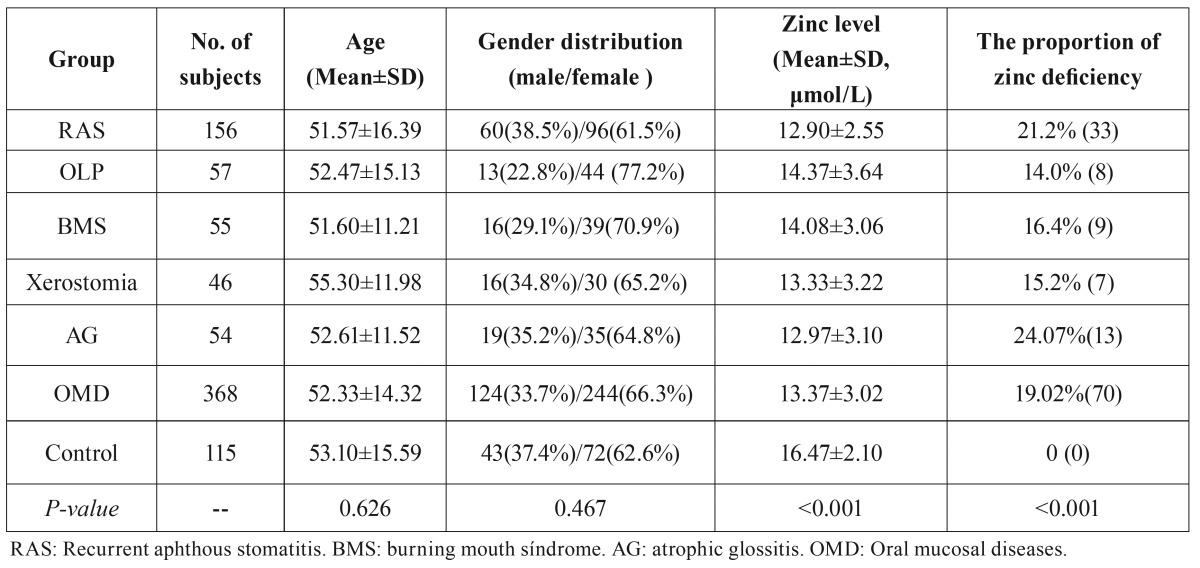
The basic parameters, zinc levels, and zinc deficiency in each group.

- Zinc deficiency condition

It is worth noting that in the healthy control group, none of the serum zinc levels fell below the minimum normal value. However, the zinc deficiency rate was 19.02% (70/368) for patients with any OMD (all OMD groups collectively). Specifically, 24.07% (13/54) of patients with AG were presented with zinc deficiency, followed by 21.2% (33/156) of patients with RAS, 16.4% (9/55) of patients with BMS, 15.2% (7/46) of patients with xerostomia, and 14.0% (5/57) of patients with OLP. Statistically significant differences were present between all OMD groups and the healthy control group (*p* <0.001,  Table 3).

- Gender differences

Interestingly, the serum zinc levels in males were higher than in females for both the control and OMD groups, with the exception of the AG group. However, these differences were not statistically significant ( Table 4).

**Table 4:**
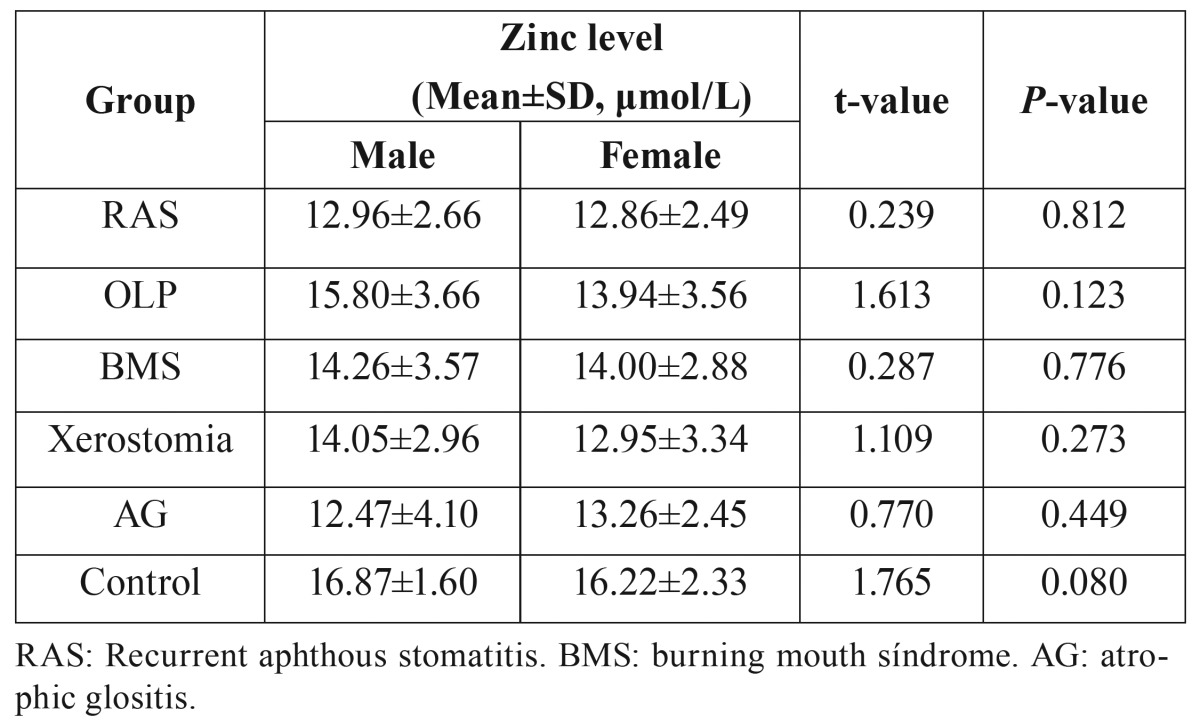
Gender differences in zinc levels in each group.

## Discussion

Zinc serves as a cofactor of at least 3000 human proteins, and it participants in various molecular mechanisms, which means that zinc deficiency may be more widely and significantly involved in the pathogenesis of human diseases than generally accepted (6,7,21). Zinc deficiency results in multiple abnormalities, which have a positive correlation with occurrence of the oral mucosal diseases discussed in this paper (Fig. 1). In this study, serum zinc levels in OMD patients were significantly lower than or were at the lower end of the normal range, which is in stark contrast to healthy patients that show no signs of zinc deficiency and have zinc levels that are in higher end of the normal zinc range. This simple observation suggests that serum zinc levels maybe influence the pathogenesis of oral mucosal diseases.

It is widely believed that atrophic glossitis (AG) may be a marker of nutritional deficiency and the significant association of deficiency of iron, vitamin B12 and hemoglobin positivity with AG has been proved (20). In the present study, zinc deficiency was found in nearly a quarter of the AG patients, compared with no deficiency in healthy control. A previous research in Norway revealed that AG was a marker for malnutrition but not related to zinc level (12). However, a more recent and larger scale study in the same Northern Europe country exhibited a relatively high prevalence of zinc deficiency in elderly people and demonstrated the significant association between zinc deficiency and the risk of malnutrition (22). The inconsistent results may be due to the lack of statistical analysis about the prevalence of zinc deficiency of the AG patients in the former study. Similar to our results, the significantly lower serum zinc level or higher prevalence of zinc deficiency was also detected in patients with RAS (9,10), OLP (14), BMS (4,11) and xerostomia (13). Further investigation is required to determine if low zinc levels are secondary to or facilitate disease development. However, we would suspect that low levels of serum zinc are a predisposing factor that may render the individual more susceptible to oral mucosal dysfunction, precipitating the occurrence of OMDs.

The observation that people without OMDs have higher/normal zinc levels, suggests that zinc homeostasis may play a role in OMD prevention and that exogenous zinc could be potentially used as a treatment or preventative measure for OMDs. Despite our findings that zinc deficiency is common in patients with OMD, additional research is needed to determine if modulation of zinc levels would be therapeutically advantageous. We speculate that zinc supplementation could be beneficial for the treatment of oral mucosal diseases. However, this viewpoint is controversial, as many patients exhibit a poor response to exogenous zinc and there may be a high incidence of undesirable side effects with systemic zinc administration. For example, Wray *et al.* (8) suggested that a routine of zinc supplementation is not advisable/beneficial for patients with RAS. But a more recent article suggested that zinc supplementation may have some positive effects (9). A study in Korea showed that zinc supplementation can be effective against BMS (4), but this view was challenged in a later publication (5). At present, the debate as to the value of zinc supplementation in patients with OMDs continues. Zinc supplementation must be carefully considered, since the optimal level of zinc in the human body is still unknown, and the potential toxic effects of zinc must be considered (21).

We demonstrated that the serum zinc level is likely lower in women than in men. Similar results have been obtained in a large-scale study performed across three European countries (23). Coincidentally, the OMD diseases discussed here are more prevalent in women than man. For example, several studies have denoted that BMS is highly associated with the female gender (24,25). In addition, OLP is twice as common in women as in men (26). Thus, we speculate that the lower level of serum zinc may be one factor that helps explain the increased prevalence of OMD in women.

Our study reveals significant differences in zinc levels between patients with OMD and healthy individuals. Therefore, we postulate that zinc deficiency and lower serum zinc levels are involved in the pathogenesis of oral mucosal diseases and specifically that a decline in a person’s serum zinc level may be predisposing factor for the development of an OMD. However, additional study is necessary to determine the efficiency and safe dosing for zinc supplementation in humans, especially considering the overt controversy surrounding this approach. With convenient accessibility and easy traceability, serum zinc levels might also serve a predictive or diagnostic factor for the occurrence of OMDs. Additional research should help to determine the ideal administration strategy and demonstrate the potential effectiveness of zinc supplementation.
